# Treatment of Epithelioid Sarcoma at the Royal Marsden Hospital

**DOI:** 10.1080/13577140310001644760

**Published:** 2003

**Authors:** L. Livi, N. Shah, F. Paiar, C. Fisher, I. Judson, E. Moskovic, M. Thomas, C. Harmer

**Affiliations:** 1 Radiotherapy Department Florence University Florence Italy; 2 Mount Vernon Cancer Centre Northwood HA6 2RN UK; 3 Sarcoma Unit Royal Marsden Hospital Fulham Road SW3 6JJ UK; 4 Department of Clinical Oncology Florence University Viale Morgagni 85 Florence 50134 Italy

## Abstract

*Purpose:* The aim of this study was to assess treatment and outcome with respect to clinical and pathological features.

*Patients and methods:* Thirty-nine patients were identified (range 7–66 years, mean 23). Initial treatment comprised local excision in 11 patients and wide excision in 14. Post-operative external beam radiotherapy was prescribed in 22 patients with a total dose of 60 Gy, delivered in two phases.

*Results:* The cause-specific survival for the entire group was 79, 63, 56 and 45% at 1, 3, 5 and 10 years, respectively. A distal limb location was associated with a better prognosis than proximal limb location (*P* = 0.04).

*Conclusions:* Our data favour treatment with wide functional excision followed by radical dose radiotherapy in attempt to minimize risk of local recurrence, especially when primary tumours are bigger than 3 cm. Our data also suggest the same treatment for local recurrence, when technically possible, to avoid amputation.


*Sarcoma*, September/December 2003, *VOL. 7, NO. 3/4, 149–152*

Taylor & Francis healthsciences

ORIGINAL ARTICLE

### Treatment of epithelioid sarcoma at the Royal Marsden Hospital

L. LIVI[1], N. SHAH[2], F. PAIAR[1], C. FISHER[3], I. JUDSON[3], E. MOSKOVIC[3], M. THOMAS[3] & C. HARMER[3]

[1]*Radiotherapy Department, Florence University, Florence, Italy,* [2]*Mount Vernon Cancer Centre, Northwood, HA6 2RN, UK,* [3]*Sarcoma Unit, Royal Marsden Hospital, Fulham Road, SW3 6JJ, UK*

**Abstract**

*Purpose:* The aim of this study was to assess treatment and outcome with respect to clinical and pathological features.
*Patients and methods:* Thirty-nine patients were identified (range 7–66 years, mean 23). Initial treatment comprised local excision in 11 patients and wide excision in 14. Post-operative external beam radiotherapy was prescribed in 22 patients with a total dose of 60 Gy, delivered in two phases.
*Results:* The cause-specific survival for the entire group was 79, 63, 56 and 45% at 1, 3, 5 and 10 years, respectively. A distal limb location was associated with a better prognosis than proximal limb location ($P = 0.04$).
*Conclusions:* Our data favour treatment with wide functional excision followed by radical dose radiotherapy in attempt to minimize risk of local recurrence, especially when primary tumours are bigger than 3 cm. Our data also suggest the same treatment for local recurrence, when technically possible, to avoid amputation.

**Key words:** *epithelioid, radiotherapy, sarcoma and surgery*

### Introduction

Epithelioid sarcoma (ES) is a rare subtype of soft tissue sarcoma (STS) first described under its current designation in 1970.[1] It is a high grade tumour which shows both epithelial and mesenchymal differentiation.[2,3]

ES is likely to be confused with a variety of benign and malignant conditions, especially a granulomatous process,[4] synovial sarcoma,[5–7] or an ulcerating squamous cell carcinoma.[4]

The peak incidence is in young adults, being rare in children and in old age. Male patients outnumber females.[8] Principal sites of involvement are the fingers, hands and forearm; it is the most common soft tissue sarcoma to involve the hand and wrist. ES may arise in either the sub-cutis or deeper tissues. When located in sub-cutis, it usually presents as a firm nodule that may be solitary or multiple. Deep-seated lesions are usually firmly attached to tendons or tendon sheaths. Because many lesions are multinodular, determination of their exact size may be difficult.[9]

Multiple recurrences, often the result of marginal resection, are a characteristic feature of this tumour. The most common sites of metastases are regional lymph nodes and lung, less frequently central nervous system and soft tissue especially over the scalp.

Prognostic factors include gender, size, depth of the tumour and the presence of metastases. Accurate information on survival and prognostic features is difficult to ascertain in this subtype of STS. The reason for this is the rarity of the disease.

Assessment and comparison of the efficacy of treatment is exceedingly difficult to judge from the literature, as reported series are small and treatment poorly described. The aim of this study was to assess treatment and outcome with respect to the clinical and pathological features.

### Methods

A retrospective review was undertaken of the Royal Marsden Hospital Sarcoma Unit database from 1978–2001 which contains 2733 patients registered with STS. Forty-nine patients were identified with ES, but 10 had diagnostic uncertainty and have

Correspondence to: Dr. L. Livi, Department of Clinical Oncology, Florence University, Viale Morgagni 85, 50134 Florence, Italy. Tel.: +39-55-4277719; Fax: +39-55-4379930; E-mail: l.livi@dfc.unifi.it

1357-714X print/1369–1643 online © 2003 Taylor & Francis Ltd
DOI: 10.1080/13577140310001644760

been excluded from further analysis. All cases had their original histopathology reviewed. Presentation, treatment, outcome and follow-up were documented in August 2001.

Thirty-nine patients were identified with a median follow-up of 82 months (range 2 months to 22 years). The mean age was 23 years (range 7–66 years) as shown in Table 1, with a male to female ratio of 2.9:1.

The site distribution of the primary lesions is shown in Table 2. Original tumour dimensions were documented in 24 patients only from clinical evaluation, radiograph or pathological reports. Initial treatment comprised incomplete excision in four, local excision in 11, wide excision in 14, radical surgery (amputation) in eight and biopsy only in two patients. No one patient underwent regional lymph node dissection.

Radical adjuvant post-operative external beam radiotherapy was prescribed in 22 patients, with a dose range from 39.6 Gy in six fractions over 6 weeks to 60 Gy in 30 fractions over 6 weeks. A single-phase generously proportioned volume was used in 10 patients. A two-phase volume comprising the whole compartment followed by a boost to the tumour bed was planned for the other 12: nine received 50 Gy in 25 fractions plus a boost of 10 Gy in five fractions over a total of 6 weeks, one received 40 Gy in 20 fractions plus a boost of 20 Gy in 10 fractions, and two received 48 Gy in 24 fractions plus a boost of 12 Gy in six fractions.

Adjuvant chemotherapy was prescribed in only one patient comprising vincristine, cyclophosphamide and doxorubicin.

### Statistical analysis

The disease-specific and overall survivals were analysed using the Kaplan–Meier method (Statistical software). The Log-rank test was used to compare survival curves. Univariate was performed to assess the significance of prognostic factors; all tests of significance were two sided. Overall survival was defined as the time from diagnosis to death from any cause. Local disease-free survival was defined as the time from diagnosis to first local recurrence. In cases where disease progressed immediately from presentation, without response to treatment, local disease-free survival was considered to be zero.

**Table 1.** *Age distribution*

| Age distribution (years) | Number |
|---|---|
| $<30$ | 18 |
| 30–40 | 11 |
| 40–50 | 4 |
| $>50$ | 6 |

**Table 2.** *Site distribution of primary tumour*

| Location of primary tumour | Number |
|---|---|
| Distal arm | 16 |
| Proximal arm | 3 |
| Distal leg | 5 |
| Proximal leg | 8 |
| Trunk | 3 |
| Vagina/penis | 3 |
| Head and neck | 1 |

### Results

At the time of analysis, 51% of patients (20/39) were dead and 49% alive (one with metastatic disease). The cause-specific survival for the entire group was 79, 63, 56 and 45% at 1, 3, 5 and 10 years, respectively, as shown in Figure 1.

As prognostic factors for cause-specific survival we analysed tumour site, tumour size and age of patients at presentation.

Distal limb location was associated with a better prognosis than proximal limb location ($P=0.04$ on univariate analysis): at time of analysis 58% of patients with distal limb tumour were alive in contrast to only 20% who had presented with a proximal limb tumour. Patients with trunk location (2/22), vaginal involvement (1/22), regional lymph node involvement (3/22), and incompletely resected bulky disease (1/22) developed distant metastases but, apart from the patients who never were local disease free, the three with positive nodes died without evidence of local recurrence.

Age at presentation did not achieve statistical significance; however, a trend was observed, with lower age conferring a better survival. At 3 years follow-up, 73% of patients aged less than 30 years were alive, in contrast to 48 and 33% for those aged 30–50 years and over 50, respectively.

Similarly a trend was observed for size of primary tumour. We documented 100% mortality for tumours larger than 6 cm at presentation in contrast to only 40% mortality for tumours smaller than 3 cm.

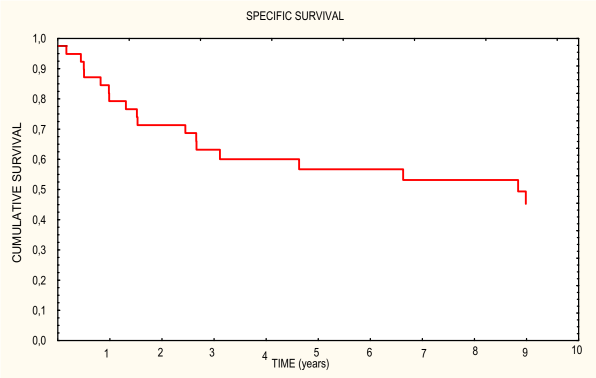

**Fig. 1.** *The cause-specific survival was 79, 63, 56 and 45% at 1, 3, 5 and 10 years, respectively.*

Twelve patients (30%) developed local recurrence (LR) as the first site of failure. The actuarial 5-year LR rate was 39%. Nine out of 12 underwent surgery only for LR and developed further local recurrence (six of whom with LR developed metastatic disease in spite of further surgery and died).

Six of the 12 patients with LR were successfully salvaged. Two underwent primary surgery only, with several local recurrences treated by surgery and eventual amputation (one patient continues to describe phantom limb pain). Three of the six underwent primary surgery only, with surgery plus adjuvant radiotherapy (50 Gy in 25 fractions + phase II 10 Gy in five fractions) for local recurrence (one subsequently required amputation for further LR). The sixth was treated by primary surgery, with surgery only for LR and died 13 years later from lung cancer but no evidence of sarcoma.

At the time of analysis, 13 patients (33%) had developed distant tumour as the first site of failure after initial therapy, at a median time of 16 months (range 4–79 months). The actuarial 5-year metastasis rate was 33%. The median post-metastasis survival was only 10 months.

Eight patients received palliative chemotherapy only, and five a combination of radiotherapy and chemotherapy. None were considered suitable for metastasesctomy because either the tumour-free interval was short or metastases were too numerous and bilateral.

### Discussion

Epithelioid sarcoma is a rare variant of STS with characteristic features both clinically and on histopathology. In most series it comprises less than 1% all of STS.[10] There is still no consensus as to the exact nature and cell type of ES.

ES has a propensity to occur in young male adults and tends to favour limb locations, especially the distal upper limb.[11,12]

Precise information on survival and prognostic features is difficult to ascertain in this rare subtype of STS. Bos *et al.*[13] report 70% survival at 5 years, with other studies quoting a range between 58 and 100%. In our experience, cause-specific survival was 63, 56 and 45% at 3, 5 and 10 years, respectively; the first local recurrence is likely to occur within the first 3 years. However, late relapse may occur: one patient developed first local recurrence after 8 years from the end of treatment and two patients developed further local recurrence after 7 years from end of second treatment.[14,15]

Metastatic disease generally occurred within 2 years after the end of treatment. When local recurrence had previously occurred, metastatic disease followed within 2 years.

In previous series, adverse prognosis has been correlated with size of the primary tumour, proximal limb and axial tumour site, depth of tumour in relation to deep fascia, and local recurrence or lymph node involvement.[16,17]

In our series, univariate analysis confirms a better prognosis for distal limb tumours ($P = 0.04$). There is no statistical significance for the other parameters, but this is likely to be a reflection of the small number of patients evaluated.

Although our results do not demonstrate statistical significance, there is a definite trend for improved disease-free survival and overall survival for surgery with clear excision margins followed by adjuvant radiotherapy. In our series, 55% of patients treated with local excision had local recurrence in contrast with 35% of patients treated with wide excision. For overall survival, mortality rates of 75, 27 and 21% are noted for patients who underwent incomplete excision, local excision and wide excision, respectively. Subgroup analysis of tumours more than 3 cm in size demonstrates mortality rates of 100, 50 and 28% for patients treated with incomplete, local and wide excision, respectively. This is supported by Callister *et al.*, who reported the multivariate analysis of the factor correlating with the overall, disease-free and metastasis-free survival: the favourable prognostic significance of small lesion size.[18]

Amputation, used either as initial primary treatment or for recurrence, did not confer a survival advantage, as previously documented.[19]

For the more common varieties of STS, adjuvant radical dose radiotherapy encompassing the entire anatomical compartment improves local control and survival. For ES, this is supported by Suit *et al.*[20,21] and Shimm & Suit[22] where a low local recurrence rate is noted.

Our study fails to demonstrate a significant advantage for adjuvant radiotherapy, but this may be confounded by the disparity of tumour sizes. The incidence of LR was similar (30%) for patients treated with adjuvant radiotherapy or surgery only; however, in the former group all tumours were bigger than 3 cm compared to the latter group where all tumours were smaller than 3 cm.

Talbert and Kinsella also confirmed improved outcome using adjuvant radiotherapy both after primary surgery and after surgery for local recurrence.[23–27]

This type of radiotherapy treatment was effective as adjuvant treatment after primary surgery and as second treatment after surgery for local recurrence.

Significant late sequelae and abnormal limb function were not observed in our series. This is supported by Okunieff *et al.*,[28] who reported that patients with local control after combined modality treatment of the hand and wrist, had less than a 25% impairment in limb function and had no pain or oedema.

The frequency of recurrence within the high-dose volume (six patients treated with a total dose of

60 Gy, four of whom received 50 Gy + 10 Gy) may suggest the need for use of a higher total dose of radiotherapy.[19]

Considering our median time for distant metastases and the possibility of LR also after several years, we recommended a follow-up every 3 months, combining clinical examination and chest X-ray for the first 3 year; then twice a year for another 2 years and then annually.

### Conclusions

Our series is too small to draw any definitive conclusion and the results must be interpreted with caution. The dominant prognostic factor was the site of the primary tumour, with best prognosis for distal limb location. Our data favour treatment with wide functional excision, followed by radical dose radiotherapy in an attempt to minimize risk of local recurrence, especially when primary tumours are bigger than 3 cm. We also recommend the same treatment for local recurrence, when technically possible, to avoid amputation which should be reserved for inoperable recurrent disease following previous radiotherapy, in the absence of distant spread.

When metastatic disease develops, prognosis is poor, with a median survival of only 10 months. Chemotherapy has not been proven to prolong survival, but novel agents and scheduling are under investigation. We recommend earlier diagnosis, dependent on increased clinical and pathological awareness, as diagnosis is typically delayed. Finally, we recommend earlier referral to a multidisciplinary specialist team within the cancer network.

### References

1. Enzinger F. Epitheloid sarcoma. A sarcoma simulating a granuloma or a carcinoma. *Cancer* 1970; 26: 1029–41.
2. Fisher C. Epithelioid sarcoma: the spectrum of ultrastructural differentiation in seven immunohistochemically defined cases. *Hum Pathol* 1988; 19: 265–75.
3. Smith ME, Brown JI, Fisher C. Epithelioid sarcoma: presence of vascular-endothelial cadherin and lack of epithelial cadherin. *Histopathology* 1998; 33: 425–31.
4. Enzinger, Weiss, eds. Malignant soft tissue sarcoma of uncertain types. In: *Soft Tissue Tumours*, Chapter 37. St. Louis, MO: Mosby, 2001; 1523–34.
5. Fletcher CDM, Beham A, Bekir S, *et al.* Epithelioid angiosarcoma of the deep soft tissue: a distinctive tumor readily mistaken for an epithelioid neoplasm. *Am J Surg Pathol* 1991; 15: 915.
6. Miettinen M, Letho VP, Vartio T, *et al.* Epithelioid sarcoma: an immonohistochemical analysis of 112 classical and variant cases and discussion of the differential diagnosis. *Hum Pathol* 1999; 30: 934.
7. Hazelbag HM, Mooi WJ, Fleurn GJ, *et al.* Gene-specific keratin profile of epithelioid soft-tissue sarcoma: an immunohistochemical, study on synovial sarcoma and epithelioid sarcoma. *Appl Immunohistochem* 1996; 4: 176.
8. Chase DR, Enzinger FM. Epithelioid sarcoma. Diagnosis, prognostic indicators, and treatment. *Am J Surg Pathol* 1985; 9: 241–63.
9. Enzinger, Weiss, eds. Malignant soft tissue sarcoma of uncertain types. In: *Soft Tissue Tumours*, Chapter 37. St. Louis, MO: Mosby, 2001; 1521–2.
10. Ross HM, Lewis JJ, Woodruff JM, Brennan MF. Epithelioid sarcoma: clinical behavior and prognostic factors of survival. *Ann Surg Oncol* 1997; 4: 491–5.
11. Spillane AJ, Thomas JM, Fisher C. Epithelioid sarcoma: the clinicopathological complexities of this rare soft tissue sarcoma. *Ann Surg Oncol* 2000; 7: 218–25.
12. Enzinger, F. Malignant soft tissue sarcoma of uncertain types. In: Enzinger F, ed. *Soft Tissue Tumours*, Chapter 38. St. Louis, MO: Mosby, 1995.
13. Bos GD, *et al.* Epithelioid sarcoma. An analysis of fifty-one cases. *J Bone Joint Surg Am* 1988; 70: 862–70.
14. Cleator SJ, Cottrill C, Harmer C. Pattern of local recurrence after conservative surgery and radiotherapy for soft tissue sarcoma. *Sarcoma* 2001; 5: 83–8.
15. Evans HL, Baer SC. Epithelioid sarcoma: a clinicopathologic and prognostic study of 26 cases. *Semin Diagn Pathol* 1993; 10: 286–91.
16. Fong Y, Coit DG, Woodruff JM, Brennan MF. Lymph node metastasis from soft tissue sarcoma in adults. Analysis of data from a prospective database of 1772 sarcoma patients. *Ann Surg* 1993; 217: 72–7.
17. Prat J, Woodruff JM, Marcove RC. Epithelioid sarcoma: an analysis of 22 cases indicating the prognostic significance of vascular invasion and regional lymph node metastasis. *Cancer* 1978; 41: 1472–87.
18. Callister MD, Ballo MT, Pisters PWT, *et al.* Epithelioid Sarcoma:results of conservative surgery and radiotherapy. *Int J Radiat Oncol Biol Phys* 2001; 51(2): 384–91.
19. Hasegawa T, *et al.* Proximal-type epithelioid sarcoma: a clinicopathologic study of 20 cases. *Mod Pathol* 2001; 14: 655–63.
20. Suit HD, Russell WO, Martin RG. Management of patients with sarcoma of soft tissue in an extremity. *Cancer* 1973; 31: 1247–55.
21. Suit HD, Russell WO, Martin RG. Sarcoma of soft tissue: clinical and histopathologic parameters and response to treatment. *Cancer* 1975; 35: 1478–83.
22. Shimm DS, Suit HD. Radiation therapy of epithelioid sarcoma. *Cancer* 1983; 52: 1022–5.
23. Jyothrmayi R, Sittamplam Y, Harmer C. Soft tissue sarcoma of the hand or foot: conservative surgery and radiotherapy. *Sarcoma* 1999; 3: 17–24.
24. Harmer C, Bidmead M. Three-dimensional planning and conformal radiotherapy. *Cancer Treat Res* 1997; 91: 129–41.
25. Whitworth PW, Pollock RE, Mansfield PF, Couture J, Romsdahl MM. Extremity epithelioid sarcoma. Amputation vs local resection. *Arch Surg* 1991; 126: 1485–9.
26. Talbert ML, Zagars GK, Sherman NE, Romsdahl MM. Conservative surgery and radiation therapy for soft tissue sarcoma of the wrist, hand, ankle, and foot. *Cancer* 1990; 66: 2482–91.
27. Kinsella TJ, Loeffler JS, Fraass BA, Tepper J. Extremity preservation by combined modality therapy in sarcomas of the hand and foot: an analysis of local control, disease free survival and functional result. *Int J Radiat Oncol Biol Phys* 1983; 9: 1115–9.
28. Okunieff P, Suit HD, Proppe KH. Extremity preservation by combined modality treatment of sarcomas of the hand and wrist. *Int J Radiat Oncol Biol Phys* 1986; 12: 1923–9.
